# Body Weight Reduction and Biochemical Parameters of the Patients After RYGB and SG Bariatric Procedures in 12-Month Observation

**DOI:** 10.1007/s11695-016-2400-0

**Published:** 2016-10-11

**Authors:** Małgorzata Szczuko, Natalia Komorniak, Monika Hoffmann, Joanna Walczak, Agata Jaroszek, Bartosz Kowalewski, Krzysztof Kaseja, Dominika Jamioł-Milc, Ewa Stachowska

**Affiliations:** 1 Department of Biochemistry and Human Nutrition, Pomeranian Medical University, Broniewskiego 24, 71-460 Szczecin, Poland; 2 Department of General and Vascular Surgery, Specialist Hospital named. prof. Alfred Sokołowski, Zdunowo, Poland

**Keywords:** Bariatric surgery, Dyslipidaemia, Fatty liver, Glucose metabolism, Obesity therapy

## Abstract

**Background:**

The aim of this study was to evaluate the effect of sleeve gastrectomy (SG) and Roux-en-Y-bypass (RYGB) on anthropometric and biochemical parameters, including changes in glucose levels, lipid profile and liver function. Drastic decrease in all lipid fractions a few weeks or months after the surgery could be regarded as favourable, but low level of HDL is an independent risk factor for heart diseases. Extreme load on the liver without preparation of the patient to the surgery can have negative consequences.

**Methods:**

The test group comprised of 40 female patients at the age of 42.96 with average body weight of 131.56 kg and BMI 46.49. Biochemical analyses were performed using calorimetric method.

**Results:**

No statistically significant differences were observed in glucose levels between the two types of procedures. The highest differences were noted for triglycerides levels, which decreased, as well as all cholesterol fractions, after RYGB, but were increasing during the first months after SG procedure. Changes in lipid profile, caused by the reduction of all lipid fractions, were more visible after RYGB. The decrease in total cholesterol directly and activity of liver enzymes after the procedure was as higher after RYGB as after SG. Increased activity of transaminases indicates significant liver overload.

**Conclusions:**

With the selection of groups of patients with similar initial parameters, it is not clear whether the differences between the two procedures when assessing the improvement of glycaemia are significant. However, due to invasive character of RYGB, liver overload lasting several months and lifelong limited absorption of nutrients, the possibility of SG procedure should be considered as a first option.

## Introduction

Obesity is an epidemic, which is a serious health and socio-economic problem both on individual and global scales. It facilitates the development of numerous diet-related diseases linked to the occurrence of metabolic syndrome. It is a state when fatty tissue comprises of more than 20 % of total body weight in men and 25 % in women, but the percentage can be higher in older people [2]. The basic method to fight obesity is the introduction of a rational diet, maintain proper dietary habits and increase physical activity. Body mass reduction using these methods is a long time process and can be ineffective in the case of people with hormonal imbalances or metabolic disorders, and additionally low level of physical activity or with genetic load [10, 27]. Bariatric surgery is the quickest and most effective means of body mass and fatty tissue reduction [17]. However, body weight loss is most pronounced in the first 3 months after the procedure, and later, the weight reduction becomes slower. The factors contributing to the body mass loss during this period are radical reduction of consumed food, physiologically induced metabolic changes (bile acids), hormonal changes (fatty tissue, intestines) and modifications in nerve signalling and in intestinal microflora [4, 9]. The influence of neurohormones produced in small intestine becomes more pronounced, and the decrease in their levels leads to the reduction of insulin synthesis and insulin resistance of the tissues [21]. Bariatric treatment, however, poses several health threats, including those linked to the complications related to the surgery itself, such as mortality during 30 days after the procedure (0.5–1.5 %), internal haemorrhage requiring reoperation (2 %), risk of occurring abscesses and leaks or venous thrombosis [1, 7, 16]. Long-term consequences are related to nutritional deficiencies, being the cause of osteoporosis or anaemia due to iron and cobalamin deficiencies, and require lifelong supplementation [11, 12, 15]. Indications for bariatric surgery in obesity treatment include high BMI—above 40 kg/cm^2^—the presence of diet-related diseases, such as high blood pressure, type 2 diabetes, metabolic syndrome, osteoarthritis, restrictive ventilatory defects and numerous unsuccessful dietetic interventions. Selection of appropriate treatment leading to body mass reduction should be rational and preceded by full information on available procedures. On the other hand, not considering any other treatments besides bariatric surgery in preventing the development of obesity-related diseases is the worst solution of all. There are clear indications (especially those related to diabetes and glycaemic control) supporting the use of RYGB. The discussion on using RYGB procedure in diabetes treatment is still on-going [3, 5, 8]. Another positive aspect corresponds to significant changes in lipid profile—a decrease in concentration of triglycerides and an increase in triglyceride-rich lipoproteins (TRLs) due to both reduced TRL clearance from the circulation and increased production by the liver (apoB-100 containing VLDLs) and intestine (apoB-48 containing chylomicrons) [8]. It seems though that, besides that improvement, after a year from the surgery, directly after the procedure and until about half a year after it, there is a deterioration in some blood biochemical parameters, including HDL, which results from increased lipolysis and insufficient intake of protein during postoperative period. Is being discussed better methods of bariatric surgery in the treatment of not only obesity but also metabolic diseases like diabetes, lipid disorders and hepatic steatosis. Opinions are strongly divided. The study compares two methods of SG and RG in these three aspects at the annual observation of patients with similar baseline characteristics.

### Aim of the Study

The objective of the study was to observe the patients and compare the effectiveness of the improvement of biochemical parameters in both types of procedures (RYGB and SG) during 12 months after the procedure. The changes occur during the first period after the procedure and should be thoroughly understood and properly interpreted to be able to precisely adjust dietary recommendations facilitating the recovery. There are few studies reporting dietary recommendations for people after bariatric treatment; however, they do not consider the need of supplementation of patients’ diets with monounsaturated fatty acids and lipotropic compounds, which prevent fatty liver disease [23]. It seems that due to increased lipolysis, being an outcome of postoperative stress and long-term fasting, a liver may require such support.

## Materials and Methods

To perform the studies, the consent of the Bioethical Commission at Pomeranian Medical University was obtained (No KB-0012/34/04/2014).

### Criteria regarding the test group

During the selection of the patients, we tried to include the patients (representing those who may be considered for bariatric surgery by NICE criteria) for whom such factors as sex, age, body weight and biochemical parameters were corresponding in both groups (RYGB and SG). The average age of 39 women in the test group was 43.7 ± 11.18. The average body weight was 131.6 kg and waist circumference 139.2 cm. All female patients under study had previous history of numerous unsuccessful attempts of losing weight using diets. Based on anthropometric measurements—body weight, height, waist and hip circumference—an average BMI and WHR indexes were determined (Table 1). A 31.25 % of women suffered from both diet-related diseased and hypothyroidism-treated pharmacologically. In 56.25 % of women, besides obesity, diet-related diseases were present, and other women were qualified for surgery due to numerous attempts to treat obesity using diets with no successful effects. Women were treated using one of the bariatric procedures—sleeve gastrectomy (SG) or Roux-en-Y-bypass (RYGB). The decision on which method was used was based on the criteria presented in the table below and personal questionnaire (Table 1).

**Table 1:** Criteria for selection of the method of bariatric treatment

Criterion	RYGB	SG
Age (years)	Younger people <30 years of age	>30 years of age
BMI (kg/m^2^)	<50	>50
Accompanying diseases	Metabolic (mainly diabetes)	Without diseases (“anti-incretin” theory of diabetes remission)
Dietary habits	“sweet eaters”	People who gorge themselves, large volume meals

The selection of patients according to age and results of anthropometric measurements was deliberate. The aim of this deliberate selection was to collect the patients who, from the point of view of the person performing the study, would provide optimal information for the goal of the study. Therefore, no statistic differences were observed between the test groups (Table 2).

**Table 2:** Characteristics of the test group of women before the surgery

Parameter	Total (SG + RYGB)	RYGB	SG
Age (years)	42.96 ± 10.45	42.97 ± 10.10	42.95 ± 10.53
Body weight (kg)	131.56 ± 14.18	130.08 ± 14.41	132.32 ± 13.88
Height (cm)	168.37 ± 8.69	168.49 ± 8.71	168.12 ± 8.75
WC (cm)	139.2 ± 14.33	138.38 ± 13.97	140.15 ± 14.98
HC (cm)	143.50 ± 13.73	142.77 ± 14.01	144.23 ± 12.87
BMI (kg/m^2^)	46.49 ± 6.78	45.82 ± 6.66	46.81 ± 7.06
WHR	1.02 ± 0.11	1.02 ± 0.10	1.02 ± 0.11

No statistically significant differences (*p* > 0.05)

## Biochemical Analyses

Blood from the patients was collected after fasting and lipid profile, and liver tests and glucose level were measured using calorimetry (Cobas Integra 400 plus Roche, Switzerland) at the Department of General and Vascular Surgery.

## Statistical Analysis

Statistical analysis was performed using STATISTICA 12.5 (Statsoft, Tulsa, Oklahoma, USA). The arithmetical mean, standard deviation and the significance of differences were calculated using ANOVA. Because most of the distributions differed from the normal distribution (Shapiro–Wilk test), further analysis involved non-parametric tests. Dependent samples test was used. The level of significance was *p* ≤ 0.05.

## Results

### Characteristics of the Test Group

The majority of women subjected to bariatric surgery lived in cities (78.12 %). The 18.75 % of women were highly educated, 56.25 % completed secondary education and 25 % obtained primary or vocational education. Among employed women, the dominating nature of work was physical (43.75 %) or mixed physical and intellectual (43.75 %). As much as 78.12 % of questioned women declared spending their free time in a passive way. Fifty per cent of patients ate between meals, mostly in stressful situations and to relax, and every tenth person snacked at night. In the test group, large differences were observed in the incidence rate of postoperative complications (vomiting, constipation, diarrhoea)—in the group after RYGB, the complications occurred in 37.5 % of patients, whereas in the group after SG, in 78.12 % of patients.

### Changes in Anthropometric Parameters

When comparing the reduction of anthropometric parameters after two bariatric procedures (RYGB and SG), the lack of significant differences was observed in body mass reduction, waist and hip circumference, and BMI and WHR (Table 3).

**Table 3:** Comparison of the effects of anthropometric parameters reduction 1 year after the surgery

Tested parameter	12 months after RYGB	12 months after SG	*p* value	Statistical significance*
Body weight (kg)	95.79 ± 15.1	94.27 ± 12.3	0.283	NS
WC (cm)	104.91 ± 16.25	103.96 ± 16.42	0.535	NS
HC (cm)	119.64 ± 14.32	119.36 ± 14.2	0.586	NS
BMI (kg/m^2^)	33.96 ± 4.97	33.69 ± 4.16	0.523	NS
WHR	0.876 ± 0.072	0.846 ± 0.071	0.055	NS
Reduction (kg)	32.27 ± 13.9	31.02 ± 12.3	0.138	NS

*No statistically significant differences were observed between two procedures

### Changes in Biochemical Parameters after RYGB

**Fig. 1:**
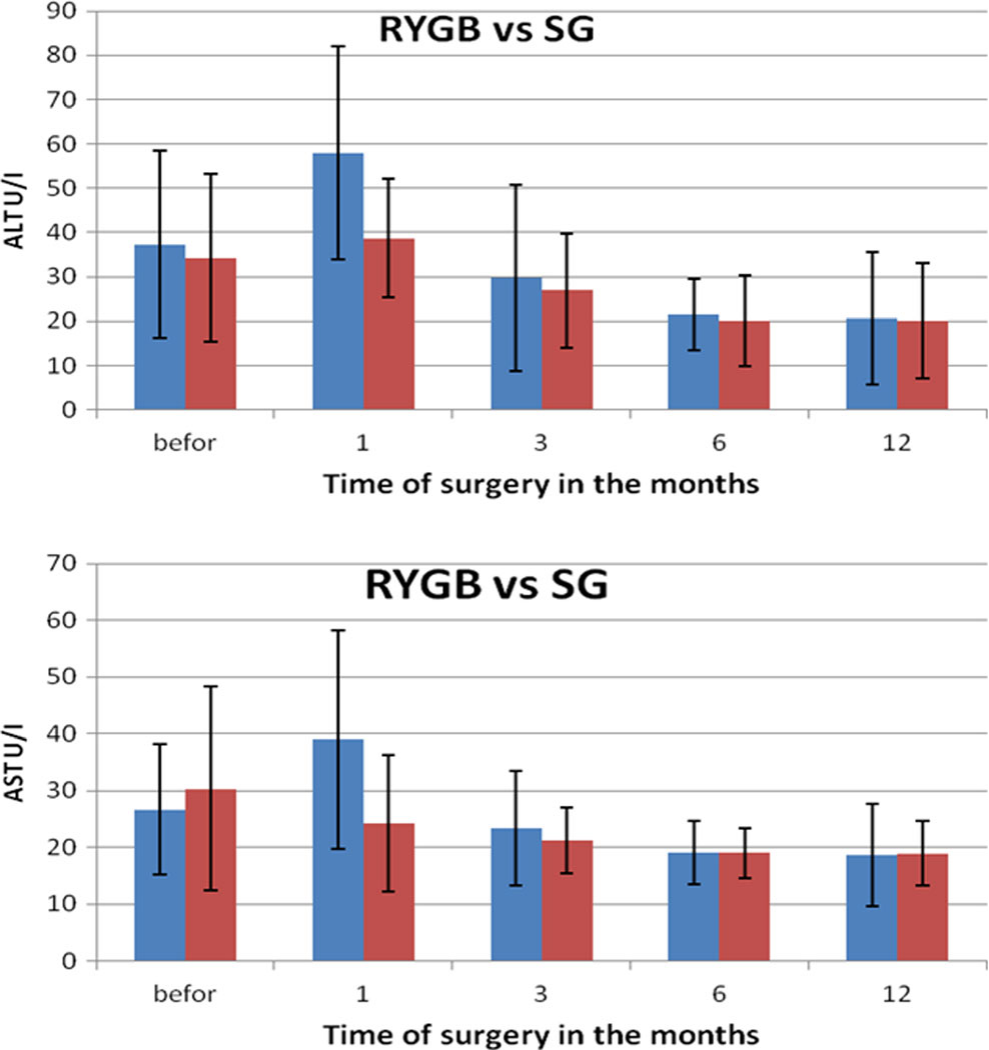
Changes in concentration of lipid fractions after bariatric surgeries performed using two methods

Changes in particular parameters in patients’ blood were different depending on the type of surgery. Before the procedure, the average concentration of cholesterol and its fractions in RYGB group amounted to total cholesterol of 200 mg/dl (SD 31.5), including TG of 148.06 mg/dl (SD 112.3), HDL of 52.36 mg/dl (SD 14.11) and LDL of 123.81 mg/dl (SD 30.3), as in Fig. 1a–d. The average level of aminotransferases was in the case of ALT 37.33 U/I (SD 21.3) and AST 26.7 U/I (SD 11.6), and the level of glucose was 108.3 mg/dl (SD 32.91).

**Fig. 2:**
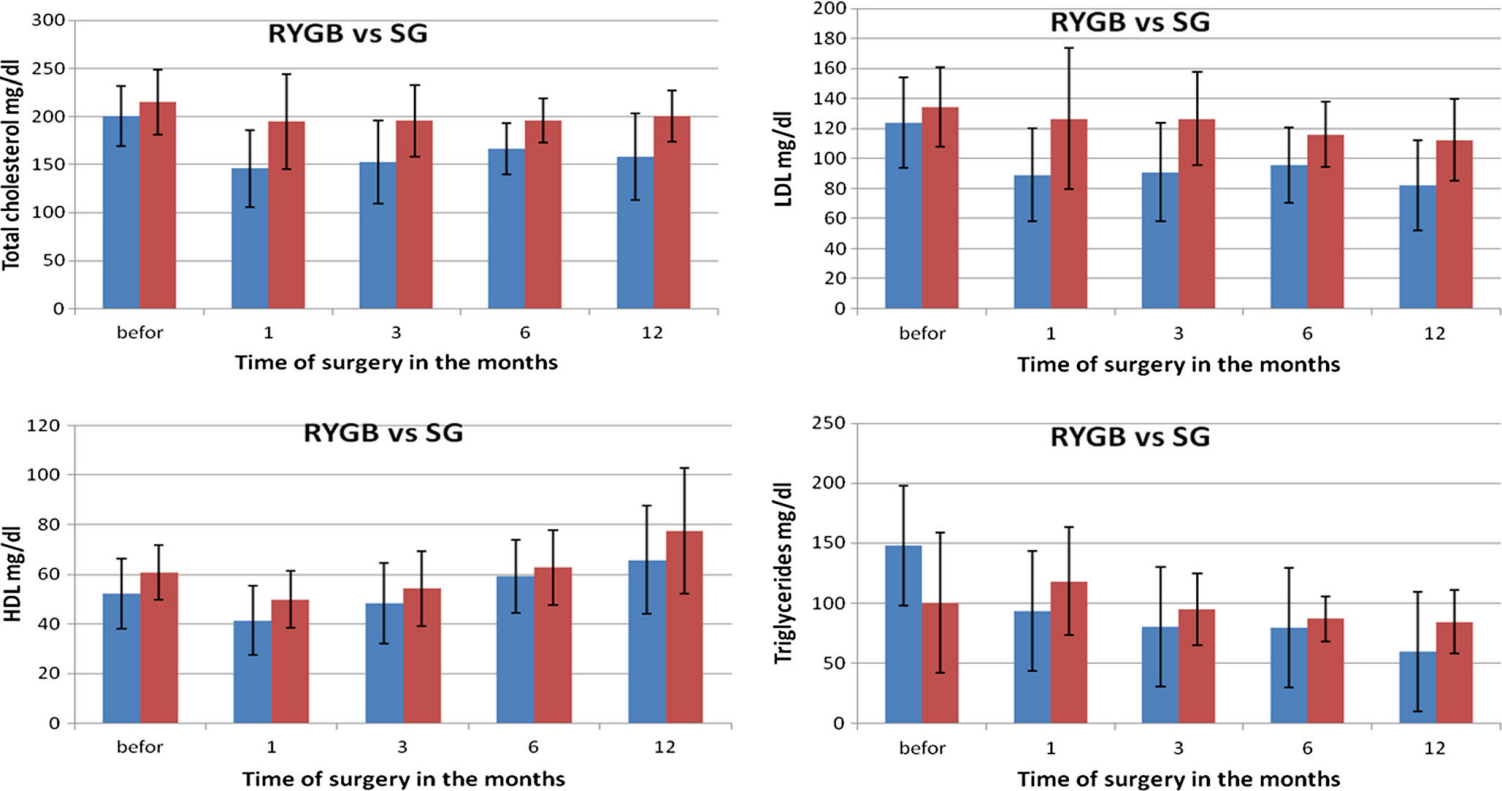
Changes in transaminases concentrations after bariatric surgeries performed using two methods

**Fig. 3:**
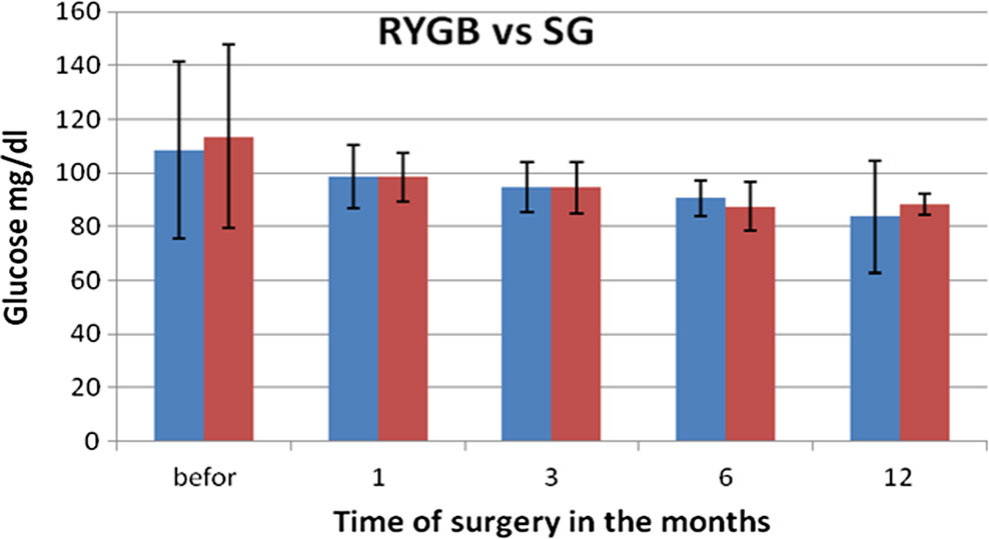
Changes in glucose concentration after bariatric surgeries performed using two methods

During a month after RYGB, a significant decrease was observed in the level of total cholesterol on average by 54.2 mg/dl, triglycerides by 54.22 mg/dl, HDL by 10.9 mg/dl and LDL by 33.73 mg/dl. These were statistically significant changes, but in the subsequent months, the levels of particular fractions fluctuated differently. The concentration of TG decreased with every measurement made every 3 months, reaching final value of 59.54 mg/dl, and similar observations were made for LDL. The level of HDL, after a period of critical reduction to 41.41 mg/dl (SD 14.02), was increasing in subsequent periods to the value of 65.73 mg/dl (SD 21.7) 1 year after the procedure. The levels of liver enzymes increased significantly 1 month after the surgery in the case of ALT and AST, and reached the levels 57.95 (SD 24.14) and 38.95 (SD 19.2), respectively. The activity of both enzymes until the 6th month after the procedure was decreasing statistically significantly, but from 6th to 12th month after the surgery, the values were stable at the level of reference values and reached 20.62 U/I (SD 15.0) for ALT and 18.67 U/I (SD 9.0) for AST (Fig. 2a, b). The level of glucose in RYGB patients was decreasing almost linearly reaching the value of 83.62 mg/dl (Fig. 3).

### Changes in Biochemical Parameters After SG

In SG group before the procedure, the average levels of total cholesterol and its fractions HDL, LDL and triglycerides amounted to, respectively, 215.03 mg/dl (SD 33.72), 60.08 mg/dl (SD 10.95), 134,34 mg/dl (SD 26.58) and 100.3 mg/dl (SD 58.14). The concentrations of aminotransferases were 34.22 mg/dl (SD 18.99) for ALT and 30.33 mg/dl (SD 17.98) for AST, and glucose level was 113.55 mg/dl (34.15).

During a month after SG procedure, a significant decrease was observed in the levels of total cholesterol by, on average, 20.31 mg/dl, HDL by 10.9 mg/dl, and LDL by 33.73 mg/dl. On the other hand, the level of triglycerides increased by 17.78 mg/dl. These changes were statistically significant. In subsequent months, the concentration of TG was decreasing, reaching the final value of 84.32 mg/dl. Similar observations were made for LDL—its final concentration reached 112.29 mg/dl. The concentration of HDL, after the period of greatest drop to 49.82 mg/dl (SD 14.02), constantly increased in further months reaching the level of 65.73 mg/dl (SD 21.7) 1 year after the surgery (Fig. 1a–d). The levels of liver enzymes were stabilized at the initial level up to 1 month after the procedure, whereas the activity of AST was decreasing. The highest changes in activity (reduction) of AST were observed in first month after the surgery and, in the case of ALT, up to 3 months after the procedure (Fig. 2a, b). Glucose level in patients in SG group, similarly as in RYGB group, decreased linearly, reaching the level of 88.12 mg/dl (Fig. 3). Statistically significant relations related to Figs. 1–3 are presented in Table 4—for parameters measured for RYGB and SG.

**Table 4:** A *p* value for biochemical parameters after RYGB and SG considering period of time

*p* value for biochemical parameters after RYGB										
Parameter	Before:1	Before:3	Before:6	Before:12	1:3	1:6	1:12	3:6	3:12	6:12
ALT	0.294	0.035*	0.008*	0.005*	0.012*	0.000*	0.000*	0.000*	0.000*	0.939
AST	0.081	0.099	0.080	0.080	0.350	0.350	0.167	0.020*	0.720	0.923
TC	0.000*	0.000*	0.028*	0.055	0.907	0.927	0.596	0.977	0.389	0.315
HDL	0.000*	0.033*	0.592	0.012*	0.022*	0.000*	0.000*	0.008*	0.000*	0.001*
LDL	0.163	0.014*	0.006*	0.000*	0.984	0.321	0.073	0.098	0.000*	0.352
TG	0.685	0.151	0.035*	0.061	0.002*	0.000*	0.000*	0.056	0.070	0.570
Glucose	0.129	0.910	0.027*	0.021*	0.210	0.007*	0.011*	0.042*	0.027*	0.140
*p* value for biochemical parameters after SG										
Parameter	Before:1	Before:3	Before:6	Before:12	1:3	1:6	1:12	3:6	3:12	6:12
ALT	0.295	0.035*	0.008*	0.005*	0.012*	0.000*	0.000*	0.000*	0.000*	0.939
AST	0.081	0.099	0.080	0.080	0.354	0.455	0.167	0.019*	0.072	0.923
TC	0.000*	0.000*	0.028*	0.055	0.901	0.927	0.595	0.977	0.389	0.316
HDL	0.000*	0.033*	0.592	0.012*	0.023*	0.000*	0.000*	0.008*	0.000*	0.001*
LDL	0.163	0.014*	0.006*	0.000*	0.984	0.321	0.073	0.098	0.000*	0.352
TG	0.683	0.151	0.035*	0.060	0.002*	0.000*	0.000*	0.057	0.070	0.570
Glucose	0.050*	0.020*	0.003*	0.004*	0.040*	0.000*	0.000*	0.011*	0.005*	0.766

*Statistically significant differences (*p* > 0.05)

### Comparison of Biochemical Parameters Between RYGB and SG 1 Year After the Surgery

Twelve months after the surgery, the reduction in transaminases activity was on comparable level in both groups. The level of total cholesterol and its fractions—LDL and triglycerides—significantly decreased in RYGB group, as compared to SG group, and HDL was on similar level in both groups (*p* = 0.112). Also, glucose concentration was not statistically significantly different between the two groups (Table 5).

**Table 5:** Comparison of biochemical parameters of female patients after both bariatric procedures (RYGB and SG) 12 months after the surgery

Tested parameter	12 months after RYGB	12 months after SG	*p* value	Statistical significance
ALT (U/I)	20.62 ± 15.01	20.00 ± 12.96	0.891	NS
AST (U/I)	18.67 ± 9.01	18.89 ± 5.64	0.928	NS
TC (mg/dl)	158.08 ± 45.00	200.51 ± 26.84	0.001	S
TG (mg/dl)	59.54 ± 24.24	84.32 ± 26.38	0.004	S
HDL (mg/dl)	65.73 ± 21.74	77.48 ± 25.04	0.112	NS
LDL (mg/dl)	82.11 ± 30.32	112.29 ± 27.34	0.023	S
Glucose (mg/dl)	83.62 ± 20.71	88.12 ± 4.01	0.419	NS

## Discussion

Sleeve gastrectomy (SG) is based on the surgical removal of ca. 70–80 % of the stomach along the greater curvature. The effect of the procedure consists in the reduction of the volume of consumed food and hunger due to decreased secretion of ghrelin and reduced nerve signalling [13]. In the method called Roux-en-Y-bypass (RYGB), the stomach is divided below the lower stomach outlet horizontally into two parts. The upper part forms smaller stomach pouch connected to small intestine loop via a “Roux limb”. Lower part remains physiologically linked to duodenum and proximal part of jejunum, through which the bile and digestive enzymes are transported. Digestion and absorption are thus limited, because they occur on a shorter distance of the gastrointestinal tract. The disadvantages of the first method are twice as frequent postoperative problems such as vomiting, constipation or diarrhoea. The disadvantages of the second method are the necessity of lifelong supplementation in vitamins and minerals and regular control of their level in the organism, as well as higher invasiveness of the surgery and, due to that, more frequent postoperative complications leading to death [7]. Interestingly, in the study of Stumpf et al., the worst impact on the tolerance of food after the procedure was observed for the least invasive method, i.e. gastric banding (out of three examined methods: gastric banding, SG and RYGB) [24]. One of the advantages of RYGB seems to be the improvement in glycaemia [5, 18]. However, in our study, with the selection of patients with similar initial parameters, the improvement in glycaemia after RYGB was not statistically significant with respect to SG. Both in our study and in the studies of other authors, the reduction in body weight measured shortly after the surgery and after longer period of time (ca. 1 year) is comparable [17]. In both bariatric procedures, a metabolic response to trauma leads to the development of signalling via neurohormonal way. Its aim is to turn on own energy reserves and supply energetic and building substrates to wound healing process [22]. Undergoing metabolic changes very quickly leads to catabolism, which has the following biological effect: lower retention of sodium ion and water in the organism, increased glucose concentration in blood (caused by glycolysis, gluconeogenesis and glycogenolysis) and reduction of pain. Catabolic effects also involve escalated degradation of protein, increased concentration of ketone bodies, loss of nitrogen with urine and negative nitrogen balance, which modulates immune system in a response to inflammatory stimuli. In the first postoperative period, the energy demand is increasing, body temperature is growing, hyperventilation occurs and the energy is crucial to sustain proper tissue perfusion, ATP and phosphocreatine levels, and thus proper wound healing. The study of Tam et al. shows, however, that the resting energy expenditure (REE) adaptation mechanism after both types of surgeries (SG and RYGB) does not contribute to body mass reduction, because REE lowers by 130–300 kcal/day and these are long-term changes—measurable even 2 years after the surgery [25].

The concentrations of all lipid fractions of the serum decreased. Lipids are transported by apolipoproteins, and when the system becomes defective, the concentration of lipids in serum increases. In our case, we have an opposite situation, which is probably caused by two unrelated mechanisms. First of them, confirmed in the literature, relates to the decrease in insulin concentration and better glycaemic control [9, 18]. Lower insulin levels lead to increased secretion of fatty acids from fatty tissue. In principle, insulin inhibits lipase in fatty tissue; therefore, lower concentration of insulin facilitates the release of fatty acids and glycerol [20]. Therefore, we should observe the increased concentration of fatty acids in blood serum. It was also shown that reduction in insulin level is accompanied by the decrease in the activity of pyruvate dehydrogenase, acetyl-CoA carboxylase and glycerol*-*3*-*phosphate acyltransferase. Lower activity of these enzymes results in reduced synthesis of cholesterol and, consequently, steroid hormones.

During postoperative period, besides the mechanisms outlined above, there is also long-lasting fasting period resulting from restrictive dietary requirements [14]. Low intake of food leads to a distortion of signals from the intestines stimulating transporters synthesis. During fasting, a much larger amount of fat is burned than it would appear based on the amount of oxidized free fatty acids. It seems that such a large reduction in all serum lipid fractions during the 3 months after the surgery is caused by similarly low concentration of apolipoproteins transporting cholesterol esters. The synthesis of apolipoproteins and phospholipids is blocked. Reduced synthesis of phospholipids is caused by the lack of choline, which is not present in bariatric patient’s diet due to the elimination or drastic limitation of the consumption of meat, especially liver, eggs and wholegrain products. It can be expected that endogenous production of choline by intestinal bacterial flora is also stopped due to changes in microbiota [9, 26]. Additionally, lipid synthesis is affected by the deficiency in essential fatty acids, which also influences the reduction of HDL, so their supplementation in a diet should be considered. During fasting, ketone bodies become the major source of energy instead of glucose, which leads to the reduction in glucose demand. Nevertheless, small amounts of glucose are necessary and are synthesized from amino acids, coming from degraded muscle proteins and from small amounts consumed with food. It seems that surgery using SG procedure, due to its character, can cause more severe protein malabsorption in the stomach. If lower differences in the reduction of particular lipid fractions were shown for the patients after RYGB, it could prove this thesis. However, we were unable to observe such relation in our study.

Referring to our hypothesis related to fatty liver after bariatric surgery, we can definitely observe that liver becomes overloaded, because even though the concentration of alanine aminotransferase (ALT) and aspartate aminotransferase (ASP) did not increase statistically significantly, they increased considerably—by more than 10 %. Therefore, it would be advisable to supplement the diet with pyridoxal phosphate, a cofactor of transaminases, and orotic acid, which lowers the concentration of lipoproteins containing apo B. Various reports in the literature concerning lipid profile in bariatric patients indicate that the stabilization of lipid profile occurs after 12 months [6]. There are also reports showing on its significant improvement 2 years after the surgery in 92.3 % of patients and unchanged level of HDL [19]. Corwell et al. also observed that 3 months after the procedure, the level of triglycerides had decreased, but in contradiction to other studies, they claimed that HDL was increasing starting from 1 year after the surgery.

Bariatric surgeries using gastric bypass (RYGB) and sleeve gastrectomy (SG) improved the tolerance of glucose in female patients after fasting. Improvement of glucose tolerance after bariatric treatment suggests that these effects may contribute to the decrease of cardiovascular mortality after surgery. With the selection of groups of patients with similar initial parameters, the significance of differences between the two procedures when assessing the improvement of glycaemia is not clear.

The improvement in lipid profile, manifested in reduced concentration of LDL, triglycerides and total cholesterol, visible after both surgeries, and very low concentration of HDL fraction 3 months after the procedures, clearly shows on liver overload. When preparing the patient to the surgery, it seems crucial to introduce the diet supplying lipotropic compounds, complete protein and monounsaturated fatty acids. To increase the level of HDL before the surgery and to lower the risk of its dangerous reduction, the patients should consider daily physical activity few weeks before the surgery.

It may seem that the recommended bariatric surgery (due to better effects on lowering cholesterol fractions and less frequent complications) would be RYGB. However, due to its invasive character, liver overload lasting several months and lifelong limited absorption of nutrients, the possibility of SG procedure should be considered as a first option.
